# Progress Toward Measles Elimination — Worldwide, 2000–2023

**DOI:** 10.15585/mmwr.mm7345a4

**Published:** 2024-11-14

**Authors:** Anna A. Minta, Matt Ferrari, Sebastien Antoni, Brian Lambert, Takudzwa S. Sayi, Christopher H. Hsu, Claudia Steulet, Marta Gacic-Dobo, Paul A. Rota, Mick N. Mulders, Alice Wimmer, Anindya Sekhar Bose, Patrick O’Connor, Natasha S. Crowcroft

**Affiliations:** ^1^Immunization, Vaccines, and Biologicals, World Health Organization, Geneva, Switzerland; ^2^Center for Infectious Disease Dynamics, Pennsylvania State University, University Park, Pennsylvania; ^3^Global Immunization Division, Global Health Center, CDC; ^4^Division of Viral Diseases, National Center for Immunization and Respiratory Diseases, CDC.

**Summary**

**What is already known about this topic?**

Measles vaccination is effective at preventing measles, a highly contagious disease that can cause severe complications and death and requires high population immunity to interrupt transmission.

**What is added by this report?**

During 2000–2023, measles vaccination saved an estimated 60 million lives. From 2022 to 2023, coverage with the first dose of measles-containing vaccine (MCV) remained at 83%, estimated measles cases increased 20%, and the number of countries affected by large or disruptive outbreaks increased from 36 to 57. Coverage was lower and measles incidence was higher in low-income countries and countries with fragile, conflict-affected, and vulnerable settings.

**What are the implications for public health practice?**

Progress toward eliminating measles will require strengthened surveillance and urgent and targeted improvements in coverage to reach all children with 2 MCV doses.

## Abstract

Measles vaccination effectively prevents measles, a highly contagious disease that can cause severe complications and death and requires high population immunity to interrupt transmission. This report describes measles elimination progress during 2000–2023. During 2000–2023, an estimated 60.3 million measles deaths were averted by vaccination. However, despite commitment from all six World Health Organization regions to eliminate measles, no region has successfully achieved and maintained measles elimination as of the end of 2023. During the COVID-19 pandemic, estimated global coverage with the first dose of measles-containing vaccine (MCV1) declined to 81%, the lowest level since 2008. MCV1 coverage improved to 83% in 2022 but was unchanged in 2023. From 2022 to 2023, estimated measles cases increased 20% worldwide, from 8,645,000 to 10,341,000; the number of countries experiencing large or disruptive outbreaks increased from 36 to 57. Estimated measles deaths decreased 8%, from 116,800 in 2022 to 107,500 in 2023, primarily because an increased number of cases occurred in countries with lower risk for death. The stagnation in MCV1 coverage means millions of children remain unprotected, leading to increases in cases and outbreaks. Coverage with measles-containing vaccine (MCV) is lower, and measles incidence is higher, in low-income countries and countries experiencing fragile, conflict-affected, and vulnerable settings, which exacerbate inequities. Urgent and targeted efforts are needed to ensure that all children receive 2 MCV doses and that surveillance is strengthened to hasten progress toward measles elimination.

## Introduction

Measles is a highly contagious disease that can cause severe complications and death (*1*). Measles vaccination is highly effective at preventing measles and, during the past 50 years, has saved an estimated 94 million lives (*2*). Although all countries in the six World Health Organization (WHO) regions have committed to eliminating measles,* no region has both achieved and sustained measles elimination as of the end of 2023. The Immunization Agenda 2030 (IA2030) includes measles elimination as a core indicator of impact of immunization programs, highlighting the importance of rigorous measles surveillance systems to identify immunity gaps and achieving equitable 95% coverage with 2 timely doses of measles-containing vaccine (MCV) to close these gaps (*3*). Measles infections act as a tracer of the health system’s capacity to deliver essential vaccines during childhood. This report updates a previous report (*4*) and describes progress toward measles elimination during 2000–2023.

## Methods

### Immunization and Surveillance Data Collection and Analysis

Each year, countries report data on vaccinations delivered through routine immunization services, supplementary immunization activities (SIAs),^†^ and outbreak response activities to WHO and UNICEF through the Joint Reporting Form (JRF). WHO and UNICEF estimate coverage with first and second MCV doses (MCV1 and MCV2, respectively) delivered through routine immunization services^§^ for all countries. Countries report the number of annual incident measles cases through JRF and monthly discarded cases^¶^ to WHO. Vaccination coverage, reported measles incidence,** and discarded case rates are calculated using population estimates from the 2024 United Nations Population Division update.^††^ Countries’ vaccination and reported case data are categorized by income group^§§^; presence of fragile, conflict-affected, and vulnerable (FCV) settings^¶¶^; and presence of large or disruptive outbreaks (20 or more cases per 1 million population over 12 months). Laboratory data are generated by the Global Measles and Rubella Laboratory Network (GMRLN), which consists of 762 laboratories supporting measles and rubella surveillance by confirming cases through quality-controlled laboratory testing and performing genotyping of circulating measles viruses (*5*).

### Modeling Estimates

Because routine surveillance data typically underestimate measles cases, measles cases and deaths were estimated using a previously described model updated with 2023 measles and United Nations population estimates*** (*6*). Data on case fatality rates from a publicly available statistical package (measlesCFR)^†††^ were used in the model to calculate estimates of measles mortality based on previously published methodology (*7*). These activities were reviewed by CDC, deemed not research, and were conducted consistent with applicable federal law and CDC policy.^§§§^

## Results

### Immunization Activities

During 2000–2019, estimated MCV1 coverage increased worldwide from 71% to 86%, then declined to 81% in 2021 during the COVID-19 pandemic, increased to 83% in 2022, and remained unchanged in 2023 (Table 1). Coverage in all regions declined during 2019–2021 and only increased during 2022–2023 in the African Region (AFR), Region of the Americas (AMR), and European Region (EUR). No region regained its 2019 MCV1 coverage levels. In 2023, MCV1 coverage was 64% in low-income countries, 86% in middle-income countries, and 94% in high-income countries; it was 67% and 89% in countries with and without FCV settings, respectively (Supplementary Table 1, https://stacks.cdc.gov/view/cdc/168892). During 2023, MCV1 coverage in the 104 countries affected by at least one large or disruptive measles outbreak during 2019–2023 was 80% compared with 91% in nonaffected countries.

**TABLE 1:** Estimates of regional immunization coverage with the first and second doses of measles-containing vaccine administered through routine immunization services, reported measles cases, and reported measles incidence, by World Health Organization region — worldwide, 2000–2023

WHO region/Year (no. of countries in the region)*	Percentage				No. of reported measles cases^¶^ (% of total cases)	Measles incidence^¶,^**^,††^
	MCV1 coverage^†^	Countries with ≥95% MCV1 coverage^§^	MCV2 coverage^†^	Reporting countries with fewer than five measles cases per 1 million population^¶,^**		
**Total (all regions)**						
**2000 (191)**	**71**	**28**	**17**	**33**	**853,479 (100.0)**	**144.6**
**2016 (194)**	**85**	**42**	**67**	**65**	**132,490 (100.0)**	**18.0**
**2019 (194)**	**86**	**44**	**71**	**44**	**873,373 (100.0)**	**118.8**
**2020 (194)**	**83**	**30**	**71**	**58**	**159,073 (100.0)**	**21.2**
**2021 (194)**	**81**	**30**	**71**	**68**	**123,171 (100.0)**	**16.4**
**2022 (194)**	**83**	**34**	**73**	**61**	**205,173 (100.0)**	**28.0**
**2023 (194)**	**83**	**35**	**74**	**47**	**663,795 (100.0)**	**91.0**
**African**						
2000 (46)	53	2	5	6	**520,102 (60.9)**	821.3
2016 (47)	68	15	22	49	**36,269 (27.4)**	35.9
2019 (47)	71	13	33	34	**618,595 (70.8)**	551.8
2020 (47)	69	6	39	30	**115,369 (72.5)**	104.8
2021 (47)	67	4	40	34	**88,789 (72.1)**	80.8
2022 (47)	68	11	44	23	**97,230 (47.4)**	80.5
2023 (47)	70	11	49	13	**424,433 (63.9)**	342.9
**Americas**						
2000 (35)	93	40	65	89	**1,754 (0.2)**	2.1
2016 (35)	92	46	80	97	**97 (0.1)**	0.1
2019 (35)	87	43	72	89	**21,971 (2.5)**	32.6
2020 (35)	86	20	73	97	**9,996 (6.3)**	9.8
2021 (35)	85	17	77	97	**682 (0.6)**	0.7
2022 (35)	84	17	76	91	**47 (—)**	0.1
2023 (35)	85	14	75	86	**14 (—)**	0
**Eastern Mediterranean**						
2000 (21)	70	29	27	14	**38,592 (4.5)**	85.8
2016 (21)	81	57	73	57	**6,275 (4.7)**	9.3
2019 (21)	82	48	75	38	**18,458 (2.1)**	26.0
2020 (21)	82	38	75	48	**6,769 (4.3)**	10.1
2021 (21)	80	43	75	52	**26,089 (21.2)**	39.2
2022 (21)	80	52	75	38	**56,401 (27.5)**	80.9
2023 (21)	79	57	73	19	**92,761 (14.0)**	122.8
**European**						
2000 (52)	91	45	47	38	**37,421 (4.4)**	49.8
2016 (53)	93	49	88	77	**4,440 (3.4)**	5.2
2019 (53)	96	60	92	30	**106,481 (12.2)**	115.0
2020 (53)	94	43	91	74	**10,945 (6.9)**	13.4
2021 (53)	95	47	91	94	**99 (0.1)**	0.1
2022 (53)	94	51	91	92	**852 (0.4)**	0.9
2023 (53)	95	55	91	64	**55,589 (8.4)**	74.7
**South-East Asia**						
2000 (10)	63	18	3	0	**78,558 (9.2)**	50.3
2016 (11)	89	55	75	27	**27,530 (20.8)**	14.0
2019 (11)	94	64	83	27	**29,389 (3.4)**	14.7
2020 (11)	88	45	80	45	**9,389 (5.9)**	4.8
2021 (11)	87	45	79	55	**6,448 (5.2)**	3.3
2022 (11)	94	45	86	64	**49,201 (24.0)**	23.6
2023 (11)	91	36	85	36	**85,368 (12.9)**	40.7
**Western Pacific**						
2000 (27)	85	30	2	26	**177,052 (20.7)**	105.6
2016 (27)	96	52	93	48	**57,879 (43.7)**	30.8
2019 (27)	96	59	93	41	**78,479 (9.0)**	40.9
2020 (27)	95	44	93	37	**6,605 (4.2)**	3.4
2021 (27)	92	41	91	56	**1,064 (0.9)**	0.6
2022 (27)	93	44	92	44	**1,442 (0.7)**	0.8
2023 (27)	92	44	90	52	**5,630 (0.9)**	3.1

**Abbreviations:** MCV1 = first dose of measles-containing vaccine; MCV2 = second dose of measles-containing vaccine; WHO = World Health Organization.

* All countries that are United Nations members can become members of WHO by accepting its constitution. Other countries can be admitted as members when their application has been approved by a simple majority vote of the World Health Assembly. https://www.who.int/countries

^†^ https://immunizationdata.who.int/ (Accessed July 19, 2024).

^§^ Denominator is the number of WHO member countries.

^¶^ https://immunizationdata.who.int/ (Accessed July 23, 2024).

** Population data from United Nations Department of Economic and Social Affairs, Population Division, 2024. Any country not reporting measles cases for that year was removed from the numerator and denominator when calculating incidence.

^††^ Cases per 1 million population.

In 2023, 22.2 million children did not receive MCV1 through routine immunization services, an increase of 472,000 (2%) compared with 2022, but a 2.1 million (9%) decrease compared with 2021. The 10 countries with the highest number of infants who did not receive MCV1 were in AFR (four countries), Eastern Mediterranean Region (EMR) (four), and South-East Asia Region (SEAR) (two), representing 57% of all children worldwide who did not receive MCV1.

During 2000–2019, estimated MCV2 coverage increased from 17% to 71%, primarily owing to MCV2 introductions. However, the increase in MCV2 coverage stalled during 2020–2021 amid the COVID-19 pandemic, then increased to 73% in 2022 and 74% in 2023. The number of countries offering MCV2 increased by 101%, from 95 (49%) of 194 countries in 2000 to 190 (98%) in 2023, including two additions in 2023. Approximately 112 million persons received MCV through SIAs in 37 countries in 2023, and another 9.4 million during measles outbreak response activities in 14 countries.

### Surveillance Performance and Reported Measles Incidence

Among the 149 (77%) countries reporting discarded cases in 2023, the measles surveillance sensitivity indicator target of two or more discarded cases per 100,000 population was achieved by 86 (58%) countries, compared with 74 (51%) of 145 countries in 2022. In 2023, GMRLN received 436,421 specimens for measles testing compared with 274,270 in 2022.

During 2023, the number of reported measles cases (663,795) increased 224% compared with cases during 2022 (205,173 cases), corresponding to a 225% increase in incidence from 28 to 91 cases per 1 million population (Table 1). In 2023, measles incidence in low-income countries was 583 per million compared with 37 and 26 per million in middle- and high-income countries, respectively. In 2023, measles incidence in countries with FCV settings was 362 cases per million, more than 10 times that in non-FCV countries (34 per million).

In 2023, large or disruptive measles outbreaks occurred in 57 countries in five WHO regions, an increase of 58% compared with 36 countries in four regions in 2022. Among the 57 outbreaks in 2023, 27 (47%) occurred in countries in AFR, 13 (23%) in EMR, 10 (18%) in EUR, four (7%) in SEAR, and three (5%) in the Western Pacific Region (WPR) (Supplementary Table 2, https://stacks.cdc.gov/view/cdc/168893).

### Reported Measles Genotypes

The number of measles genotypes reported by GMRLN has decreased, from nine in 2013 to two since 2021. In 2023, a total of 3,373 sequences from 74 countries were reported, among which 2,503 (74%) were genotype D8, and 870 (26%) were genotype B3; among 1,588 reported sequences from 48 countries in 2022, 848 (53%) were genotype D8, and 740 (47%) were genotype B3.

### Measles Cases and Mortality Estimates

On the basis of the updated model, the estimated number of measles cases decreased 72%, from 36,940,000 in 2000 to 10,341,000 in 2023; the estimated annual number of measles deaths decreased 87%, from 800,000 in 2000 to 107,500 in 2023 (Table 2). The estimated number of cases increased by 20%, and deaths decreased by 8% in 2023 compared with an estimated 8,645,000 cases and an estimated 116,800 deaths in 2022. During 2000–2023, compared with no vaccination, measles vaccination prevented an estimated 60.3 million deaths globally (Figure).

**TABLE 2:** Estimated number of measles cases and deaths,* by World Health Organization region — worldwide, 2000 and 2023

WHO region/Year (no. of countries in the region)^†^	Estimated no. (95% CI)		Change from 2000 to 2023	
	Measles cases	Measles deaths	% Estimated reduction in measles mortality	Cumulative no. of measles deaths averted by vaccination
**Total (all regions)**				
**2000 (191)**	**36,939,956 (26,084,165–51,808,643)**	**800,062 (530,300–1,140,188)**	**87**	**60,322,106**
**2023 (194)**	**10,341,059 (6,050,433–16,839,675)**	**107,482 (60,910–170,246)**		
**African**				
2000 (46)	11,475,194 (5,796,070–17,162,334)	361,694 (192,573–536,143)	79	20,959,652
2023 (47)	4,801,946 (2,708,283–7,804,959)	75,942 (42,703–120,893)		
**Americas**				
2000 (35)	8,770 (4,385–35,080)	3	96	6,217,493
2023 (35)	375 (188–1,500)	1^§^		
**Eastern Mediterranean**				
2000 (21)	4,440,048 (2,856,258–8,230,961)	141,059 (98,351–236,713)	89	9,543,661
2023 (21)	1,382,323 (516,020–2,436,395)	15,280 (5,280–28,088)		
**European**				
2000 (52)	768,811 (485,144–1,447,060)	3,397 (2,310–5,670)	93	1,492,203
2023 (53)	306,375 (141,791–993,945)	222 (89–621)		
**South-East Asia**				
2000 (10)	14,609,126 (12,106,256–17,667,700)	265,859 (214,349–327,839)	94	17,093,757
2023 (11)	2,905,680 (2,508,418–3,403,522)	14,691 (12,457–17,514)		
**Western Pacific**				
2000 (27)	5,638,007 (4,836,053–7,265,509)	28,049 (22,715–33,812)	95	5,015,340
2023 (27)	944,360 (175,733–2,199,353)	1,347 (380–3,129)		

**Abbreviation:** WHO = World Health Organization.

* The measles mortality model used to generate estimated measles cases and deaths is rerun each year using the new and revised annual WHO/UNICEF estimates of national immunization coverage data, as well as updated surveillance data.

^†^ All countries that are United Nations members can become members of WHO by accepting its constitution. Other countries can be admitted as members when their application has been approved by a simple majority vote of the World Health Assembly. https://www.who.int/countries

^§^ Estimated measles mortality rounded to 1.

**FIGURE:**
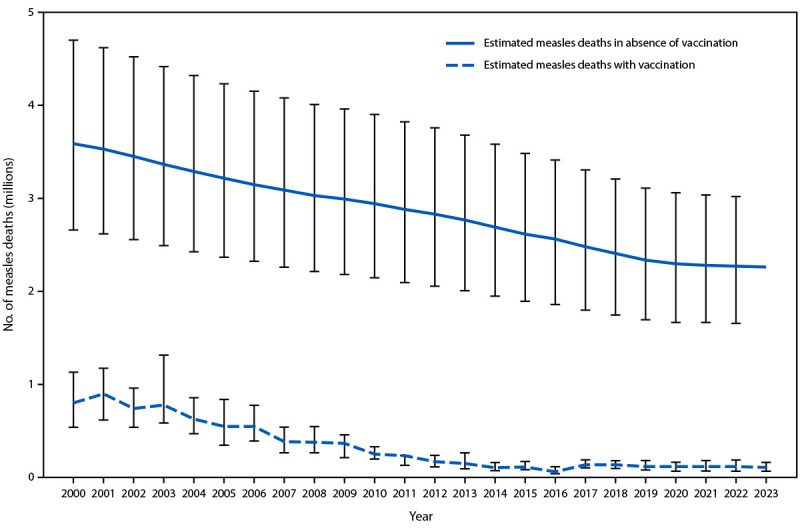
Estimated number of annual measles deaths with measles vaccination and in the absence of measles vaccination — worldwide, 2000–2023*^,^^†^ * With 95% CIs indicated by error bars. ^†^ Deaths prevented by vaccination are estimated by the area between estimated deaths with vaccination and those without vaccination. A cumulative total of 60.3 million deaths were estimated to have been prevented by measles vaccination during 2000–2023.

### Regional Verification of Measles Elimination

By the end of 2023, 82 (42%) countries had been verified to have achieved or maintained measles elimination, but no WHO region had achieved and sustained elimination, and no AFR country had yet been verified to have eliminated measles (Supplementary Table 3, https://stacks.cdc.gov/view/cdc/168894). After AMR achieved verification of measles elimination in 2016, endemic transmission was reestablished in Brazil and Venezuela; elimination was reverified in Venezuela in 2023. In November 2024, Brazil was reverified based on 2023 data, and AMR is once again free from endemic measles. 

## Discussion

Globally, MCV coverage stagnated during 2022–2023, and no region has regained pre–COVID-19 pandemic MCV1 coverage levels. AFR experienced improvements in MCV1 and MCV2 coverage in 2022–2023; however, the number of unvaccinated children will increase if coverage stagnates and cannot outpace a rapidly growing population. Given worldwide stagnant, suboptimal routine MCV1 coverage, SIAs in selected countries provide opportunities to reach children who missed routine MCV (*8*,*9*).

Measles surveillance performance has shown signs of improvement, with increasing numbers of countries achieving the target discarded case rate and increasing numbers of specimens being submitted to GMRLN for routine testing and sequencing in 2023 compared with other recent years. Improvements in the discarded case rate can be due to improved surveillance performance; however, an increase in testing in outbreak settings also contributes.

From 2022 to 2023, more countries experienced large or disruptive outbreaks, with EUR, EMR, SEAR, and WPR experiencing more large or disruptive outbreaks. The distribution of measles outbreaks across more countries, including countries where children are less likely to die from measles than in AFR countries, which had a similar number of large or disruptive outbreaks during 2022–2023, resulted in a small decrease in estimated global measles deaths in 2023 compared with 2022, despite the increased number of measles cases. Vaccination coverage is lowest, and measles incidence the highest in low-income countries and in countries affected by FCV settings. These types of inequities hinder measles elimination (*10*).

### Limitations

The findings in this report are subject to at least three limitations. First, vaccination coverage and reported cases are subject to variable data quality and potentially inaccurate estimations. Second, not all countries provide adequate SIA and outbreak response data; therefore, reported MCV doses administered might be underestimated. Finally, the modeled estimates are dependent on data input and are updated annually for the current and previous years, which can introduce uncertainty and slight differences over time; however, the estimates are within expectations given the CIs.

### Implications for Public Health Practice

The Measles and Rubella Strategic Framework (*1*), aligning with IA2030, outlines strategies countries can use to improve measles surveillance, increase routine immunization to achieve ≥95% coverage with 2 MCV doses, and strengthen outbreak preparedness and response. Activities such as the Big Catch-Up^¶¶¶^ and follow-up campaigns are designed to help close immunity gaps that developed during the COVID-19 pandemic. During the previous 50 years, vaccination has made the greatest health intervention contribution to mortality reduction, with measles vaccination contributing the most benefit (*2*). Although estimated measles deaths have decreased substantially over time and AMR has now eliminated measles, an estimated 107,500 persons died globally from this vaccine-preventable disease in 2023. Countries and global partners working together is essential to accelerate efforts to reach and sustain measles elimination.

## Footnotes

* Measles elimination is defined as the absence of endemic measles virus transmission in a region or other defined geographic area for ≥12 months in the presence of a high-quality surveillance system that meets the targets of key performance indicators. Independent regional commissions verify a country’s elimination status.

† Measles SIAs are generally conducted using two target age ranges: 1) an initial catch-up SIA targets children aged 9 months–14 years, with the aim of eliminating susceptibility to measles in the general population, and 2) periodic follow-up SIAs are conducted nationwide every 2–4 years and target all children aged 9–59 months to eliminate any measles susceptibility that has accumulated in recent birth cohorts because of low MCV coverage and to protect the estimated 2%–5% of children who did not respond to MCV1. Countries can provide additional data to WHO, and data are updated retrospectively. https://immunizationdata.who.int/ (Accessed August 29, 2024).

§ Calculated for MCV1, among children aged 1 year or, if MCV1 is given at age ≥1 year, among children aged 24 months. Calculated for MCV2 among children at the recommended age for the administration of MCV2, according to the national immunization schedule. Estimates are generated using annual administrative coverage data (the number of vaccine doses administered divided by the estimated target population), national coverage estimates, and vaccination coverage surveys. https://www.who.int/teams/immunization-vaccines-and-biologicals/immunization-analysis-and-insights/global-monitoring/immunization-coverage/who-unicef-estimates-of-national-immunization-coverage; https://immunizationdata.who.int/ (Accessed July 19, 2024).

¶ A discarded measles case is defined as a suspected case that has been investigated and determined to be neither measles nor rubella by using either 1) laboratory testing in a proficient laboratory or 2) epidemiologic linkage to a laboratory-confirmed outbreak of a communicable disease that is not measles or rubella. The discarded case rate is a measure of the sensitivity of measles surveillance; the target is two or more discarded cases per 100,000 population. https://immunizationdata.who.int/ (Accessed July 23, 2024).

** To calculate incidence, only the countries reporting data are included in the numerator and denominator. Countries do not provide WHO with their reasons for not reporting measles cases. https://immunizationdata.who.int/global/wiise-detail-page/measles-reported-cases-and-incidence (Accessed July 23, 2024).

††
https://population.un.org/wpp/Download/Standard/MostUsed/

§§
https://datahelpdesk.worldbank.org/knowledgebase/articles/906519-world-bank-country-and-lending-groups

¶¶ FCV settings is a broad term describing a range of situations including humanitarian crises, protracted emergencies, and armed conflicts. https://www.who.int/teams/integrated-health-services/quality-of-care/quality-of-care-in-fragile-conflict-affected-and-vulnerable-settings; https://gho.unocha.org/

*** State-space model of unobserved measles incidence during 2000–2023 generated using the following data from all WHO countries: 1) total annual reported measles cases; 2) annual MCV1 coverage from WHO and UNICEF estimates of national immunization coverage (WUENIC); 3) annual MCV2 coverage from WUENIC; 4) annual SIAs, with coverage and age targets (subnational SIAs are discounted by the proportion of the total population targeted); 5) total annual population size; 6) total annual births; and 7) list of all countries and years for which reporting was enhanced.

††† The measlesCFR model (https://github.com/Measles-Case-Fatality-Ratio-Estimation/measlesCFR) fitted the reported case fatality ratios from a systematic review as a function of the following covariates: 1) gross domestic product per capita, 2) HIV prevalence, 3) maternal education, 4) MCV1 coverage, 5) proportion urban, 6) total fertility rate, 7) mortality rate among children aged <5 years, 8) prevalence of vitamin A deficiency, 9) war and terrorism mortality rate, 10) wasting (weight-for-height ≥1 standard deviation below the reference median) prevalence (https://www.healthdata.org/research-analysis/diseases-injuries-risks/factsheets/2021-child-wasting-level-4-risk), and 11) measles incidence. Annual measles incidence for each country and year was based on this fitted state-space model. High-income countries were excluded from this analysis.

§§§ 45 C.F.R. part 46.102(l)(2), 21 C.F.R. part 56; 42 U.S.C. Sect. 241(d); 5 U.S.C. Sect. 552a; 44 U.S.C. Sect. 3501 et seq.

¶¶¶
https://www.who.int/publications/i/item/9789240075511
